# Predictors of conversion to psychosis and mortality in first-episode substance-induced psychosis: a nationwide register-based study in South Korea

**DOI:** 10.1038/s41537-026-00760-z

**Published:** 2026-05-06

**Authors:** Yan-Hong Piao, Thi-Hung Le, Ling Li, Ariana Setiani, Soyolsaikhan Odkhuu, Woo-Sung Kim, Shahida Nazir, Yong-Suk Jang, Young-Chul Chung

**Affiliations:** 1 Department of Psychiatry, Jeonbuk National University Medical School, Jeonju, Republic of Korea; 2 Research Institute of Clinical Medicine of Jeonbuk National University, Biomedical Research Institute of Jeonbuk National University Hospital, Jeonju, Republic of Korea; 3 Department of Molecular Biology, Jeonbuk National University, Jeonju, Republic of Korea; 4 Department of Psychiatry, Jeonbuk National University Hospital, Jeonju, Republic of Korea

**Keywords:** Psychosis, Schizophrenia

## Abstract

**Objective:**

Substance-induced psychosis (SIP) is associated with a substantial risk of progression to schizophrenia spectrum disorders (SSD), bipolar disorder (BD), and premature mortality. However, evidence from Asia, particularly South Korea (SK), remains limited.

**Methods:**

We conducted a retrospective, nationwide, population-based cohort study using the Korean National Health Insurance Claims Database (2003–2012) with follow-up through 2017. Individuals aged 18–60 years with a first episode of SIP (FE-SIP) were identified after excluding those with ICD-10 codes for substance use disorders (SUD; F10-F19) and other neuropsychiatric disorders (F00–F09, F20–F29, F30.x, F31.x, F32.3, or F33.3) during a 1-year washout. Incidence was estimated using national census data. Conversion to first-episode psychosis (FEP-SSD or -BD) was examined using cumulative incidence function and Cox regression. Standardized mortality ratios (SMRs) were calculated against the general population and a SUD reference cohort.

**Results:**

A total of 2244 individuals with FE-SIP were identified. The average annual incidence proportion was 0.7 per 100,000, peaking in 2005. Over a median follow-up of 9.3 years, 655 individuals (33.2%) converted to FEP, with cumulative conversion rates of 23.3% at 5 years, and 28.8% at 10 years. Younger age, female sex, disability, longer initial hospitalization, and use of other stimulants were significant predictors of conversion. All-cause mortality was markedly elevated (SMR 33.3 vs general population; SMR 1.49 vs SUD cohort), particularly among young men.

**Conclusions:**

Despite a low incidence of FE-SIP in SK, the risks of conversion to FE-SSD/FE-BD and premature mortality are substantial. Findings underscore the need for early detection, psychoeducation, and integrated care tailored to high-risk individuals, especially within the first two years after diagnosis.

## Introduction

Substance-induced psychosis (SIP) is a disorder defined by the presence of delusions or hallucinations that develop during or soon after substance ingestion and resolve within one month or one to six months^1^ after the last exposure to the implicated substance. SIP is an important clinical diagnosis, as 21%^2^ to 25%^3^ of individuals with SIP later convert to schizophrenia (SZ) or schizophrenia spectrum disorders (SSD), and these individuals are also at high risk of suicidal attempts during follow-up^4^. However, there is paucity of research in the area of SIP particularly from Asia.

In Western countries, reported annual incidence proportion of SIP were 6.5^5^, 9.3 to 14.1^6^ 100,000 persons per year and mean incidence rate of SIP was around 13 per 100,000 person-years (PY)^7^ On the other hand, mean incidence rate in Taiwan was much lower, with 3.09 per 100,000 PY^8^ and incidence proportion over a period of 13 years in India was 1.4% (74/5257)^9^. Methamphetamine (MA) abuse continues in East Asia and shows an increasing trend among youths in South Korea, Japan, and China^10^. As the treatment of obesity has become a contemporary social issue in Korea, phentermine or phendimetrazine, which are amphetamine analogues and anti-obesity drugs, are being widely prescribed and numerous cases of psychosis induced by these drugs have been reported^11–15^. Hence, we hypothesized that the types of substances involved in SIP in Korea may differ, and that differences in substance profiles may contribute to heterogeneous conversion risks during follow-up.

Using nationwide registry database, landmark studies reported cumulative conversion rates of 27.6% and 26% to SSD or SZ, and 4.5% or 8.4% to bipolar disorder (BD) at 6-year^16^ and 20-year follow-up^17^, respectively. Only one study from Asia reported conversion rates of 22.5% for SSD and 24.3% for BD at a 15-year follow-up^8^. Risk factors related to conversion to schizophrenia spectrum disorders, including schizophrenia, include male sex, young age, impaired premorbid social function, self harm episodes, preexisting psychiatric disorders, and a higher familial risk of psychosis^3,9,17,18^. How these risk factors differ in Asian populations, particularly in South Korea (SK), remains unclear. Regardless of conversion, FE-SIP is strongly associated with increased mortality^19^. Given that SK has had the highest suicide rate for the period 2003–2019 among OECD countries^20^, further investigation of mortality in FE-SIP is warranted.

Given these knowledge gaps, we conducted a retrospective nationwide population-based cohort study using data from the Korean National Health Insurance (KNHI) claims database. Our objectives were to estimate the incidence of FE-SIP, evaluate its long-term conversion rates to FE-SSD and FE-BD, and examine all-cause mortality. We also sought to identify key sociodemographic and clinical risk factors for conversion. This study aimed to provide a much-needed regional perspective on FE-SIP and inform clinical guidelines for early intervention and integrated care within the unique context of East Asia.

## Methods

### Data source

This retrospective population-based cohort study used data from the KNHI Claims Database, managed by the National Health Insurance Service (NHIS) of SK. The database provides comprehensive information on sociodemographic characteristics, healthcare utilization, and diagnostic codes based on the 10th revision of the International Classification of Diseases (ICD-10). The mortality data were obtained from the Korean Statistical Information Service (KOSIS, https://kosis.kr).

### Study population

We identified individuals aged 18 to 60 years who received a principal diagnosis of FE-SIP between January 1, 2003, and December 31, 2012. FE-SIP was defined according to ICD-10 diagnostic codes F10.5–F19.5, which include psychotic disorders induced by alcohol, opioids, cannabinoids, sedatives, cocaine, other stimulants, hallucinogens, tobacco, volatile solvents, and multiple drug use. To reduce diagnostic misclassification, we included outpatients only if they had ≥3 consecutive encounters coded with the same FE-SIP diagnosis; inpatients were included with a single inpatient FE-SIP diagnosis.

To ensure only incident cases were captured, we applied a 12-month washout period and excluded individuals with any ICD-10 codes of F00–F09 (organic mental disorders), F10–F19 (mental and behavioral disorders due to psychoactive substance use), F20–F29 (schizophrenia, schizotypal and delusional disorders), F30.x (manic episode), F31.x (bipolar affective disorder), F32.3 (severe depressive episode with psychotic symptoms), or F33.3 (recurrent depressive disorder, current episode with psychotic symptoms) recorded in any diagnosis position within one year prior to the SIP diagnosis. The rationale for excluding individuals with substance use disorders (SUD; F10–F19) was that, in this population, mild and transient psychotic symptoms are often underreported and therefore not detected by treating clinicians. Accordingly, we assumed that this stringent criterion would allow for the identification of more genuine cases of FE-SIP. The index date was defined as the date of the first qualifying SIP diagnosis (Fig. S1).

### Outcomes

The incidence proportion of FE-SIP was calculated as the number of newly identified cases per 100,000 individuals based on population data from KOSIS. We extracted the following sociodemographic data for the study population: age, sex, type of health insurance, health insurance income quintile, residential region, and psychiatric service use other than F00–F09, F10–F19, F20–F29, F30.x, F31.x, F32.3, or F33.3. Physical illnesses were defined as the presence of any of the 17 comorbidity categories included in the Charlson Comorbidity Index during the six months before the index date, along with disability status and length of first hospitalization.

All study individuals with FE-SIP were followed from the index date until date of event (transition to first episode psychosis [FEP]), death or study end date (December 31, 2017) (see SF1). FEP was defined as the first main diagnosis of SSD (ICD-10 codes F20 to F29, excluding F21) or BD (ICD-10 codes F30-F31). To identify risk factors for conversion to FEP, FE-SSD, and FE-BD, Cox proportional hazards models were used. For the analysis of conversion to FEP, individuals who died or reached the end of the study period without conversion were censored. For the analyses of conversion to FE-SSD and FE-BD, individuals were censored at death, at the end of the study period, or at conversion to the alternative outcome (i.e., FE-BD in the FE-SSD model and FE-SSD in the FE-BD model). The proportional hazards assumption was assessed using Schoenfeld residuals. To estimate the cumulative conversion rate from FE-SIP to FEP, we used the cumulative incidence function, treating death as a competing risk. For the cumulative conversion rate from FE-SIP to FE-SSD and from FE-SIP to FE-BD, conversion to the alternative outcome and death were treated as competing risks.

Standardized mortality ratios (SMRs) were calculated by comparing observed deaths in the FE-SIP cohort with expected deaths derived from two reference populations: (1) the general Korean population aged 20–59 years in 2015–2016, and (2) individuals diagnosed with substance use disorder (SUD) aged 18–60 years (ICD-10 F10–F19, excluding F10.5–F19.5 and F10.7–F19.7) between 2003 and 2012 with follow-up through December 2017. For the general population, age categories were slightly mismatched because Statistics Korea provides mortality data in 5-year age bands. Mortality rates from the midpoint year of the study period were used as age- and sex-specific mortality rates changed only gradually during the study period^21^. We computed 95% CIs for SMRs using a Poisson distribution.

### Statistical analyses

To analyze sociodemographic characteristics, we used independent t-tests for continuous variables and Chi-square tests for categorical variables. Statistical significance was defined as a two-tailed *p* value less than 0.05. All analyses were conducted using SAS software, version 7.1, provided by the NHIS. This study followed the STROBE reporting guidelines (Supplementary Information).

### Ethical considerations

According to national laws in SK, patient registry data can be used for research purposes without approval from an institutional review board. All analyses were conducted without personal identification numbers, and only statistical results were obtained from the institutions.

## Results

### Study cohort and baseline characteristics

A total of 2244 individuals with a first-episode substance-induced psychosis (FE-SIP) were identified as the final cohort, as detailed in Fig. S1. The cohort was predominantly male (83.34%), and more than 70% of participants were over 40 years old at the time of diagnosis. As shown in Table 1, the most commonly implicated substance among men was alcohol (92.91%), while “other stimulants” were the third most common substance among women (2.14%).

**Table 1:** Sociodemographic characteristics of individuals with first-episode substance-induced psychosis.

Variables	Total (*n* = 2244)	Male (*n* = 1871)	Female (*n* = 373)	*p*
Age				<0.0001
≤30	153 (6.82)	88 (4.69)	65 (17.43)	
31–40	469 (20.90)	359 (19.15)	110 (29.49)	
41–50	919 (40.95)	797 (42.51)	122 (32.71)	
51–60	703 (31.33)	627 (33.44)	76 (20.38)	
Types of insurance				<0.0001
Employee insured	628 (27.99)	497 (26.51)	131 (35.12)	
Self-employed insured	531 (23.66)	477 (25.44)	54 (14.48)	
Medical aid	1085 (48.35)	897 (47.84)	188 (50.40)	
Health insurance quintiles				0.0220
Q1 (highest)	115 (5.12)	88 (4.69)	27 (7.24)	
Q2	276 (12.30)	220 (11.73)	56 (15.01)	
Q3	668 (29.77)	549 (29.28)	119 (31.90)	
Q4	447 (19.92)	382 (20.37)	65 (17.43)	
Q5 (lowest)	718 (32.00)	617 (32.91)	101 (27.08)	
Unknown	20 (0.89)	15 (0.80)	5 (1.34)	
Region				0.0640
Urban	1989 (88.64)	1648 (87.89)	341 (91.42)	
Rural	255 (11.36)	223 (11.89)	32 (8.58)	
Psychiatric service during past 1-year				<0.0001
Yes	510 (22.73)	364 (19.41)	146 (39.14)	
No	1734 (77.27)	1507 (80.37)	227 (60.86)	
Physical illnesses		<0.0001		
Yes	1298 (57.84)	1119 (59.68)	179 (47.99)	
No	946 (42.16)	752 (40.11)	194 (52.01)	
Specific physical disease				
Cardiac disease	151 (6.73)	125 (6.68)	26 (6.97)	0.8385
Cerebrovascular disease	91 (4.06)	81 (4.33)	10 (2.68)	0.1406
Chronic kidney disease	9 (0.40)	4 (0.21)	5 (1.34)	0.0017
Diabetes mellitus	392 (17.47)	351 (18.76)	41 (10.99)	0.0003
Hypertensive disease	413 (18.40)	360 (19.24)	53 (14.21)	0.0220
Neoplasms	60 (2.67)	52 (2.78)	8 (2.14)	0.4879
Thyroid disease	90 (4.01)	49 (2.62)	41 (10.99)	<0.0001
Tuberculosis	50 (2.23)	46 (2.46)	4 (1.07)	0.0977
Viral hepatitis, Liver disease	971 (43.27)	862 (46.07)	109 (29.22)	<0.0001
Disability				0.2830
Yes	310 (13.81)	265 (14.13)	45 (12.06)	Yes
No	1934 (86.19)	1606 (85.65)	328 (87.94)	No
Disability type		0.0570		
Brain injury	19 (6.13)	15 (5.66)	4 (1.51)	
Epilepsy	4 (1.29)	3 (1.13)	1 (0.38)	
Hearing impairment	19 (6.13)	15 (5.66)	4 (1.51)	
Hepatic disability	5 (1.61)	5 (1.89)	----	
Intellectual disability	28 (9.03)	22 (8.30)	6 (2.26)	
Kidney disease	2 (0.65)	1 (0.38)	1 (0.38)	
Mental disability	33 (10.65)	23 (8.68)	10 (3.77)	
Physical disability	165 (53.23)	147 (55.47)	18 (72.00)	
Respiratory impairment	3 (0.97)	3 (1.13)	----	
Speech impairment	3 (0.97)	3 (1.13)	----	
Visually impairment	29 (9.35)	28 (10.57)	1 (0.38)	
No	1934 (86.19)	1606 (85.65)	328 (87.94)	
Types of substance				<0.0001
Alcohol	2019 (89.97)	1742 (92.91)	277 (74.26)	
Opioids	17 (0.76)	11 (0.59)	6 (1.61)	
Cannabinoids	4 (0.18)	----	4 (1.07)	
Sedatives	12 (0.53)	5 (0.27)	7 (1.88)	
Cocaine	2 (0.09)	1 (0.05)	1 (0.27)	
Other stimulants	22 (0.98)	14 (0.75)	8 (2.14)	
Hallucinogens	29 (1.29)	23 (1.23)	6 (1.61)	
Tobacco	30 (1.34)	25 (1.33)	5 (1.34)	
Volatile solvents	16 (0.71)	12 (0.64)	4 (1.07)	
Multiple drug use	93 (4.14)	38 (2.03)	55 (14.75)	
Length of first hospitalization	9.24 ± 0.34	9.58 ± 0.30	7.53 ± 0.54	0.0255

Length of first hospitalization are presented as means ± SE; Categorical variables are presented as number (%).

*Q* Quintile.

### Incidence of FE-SIP and cumulative conversion rates

**Fig. 1:**
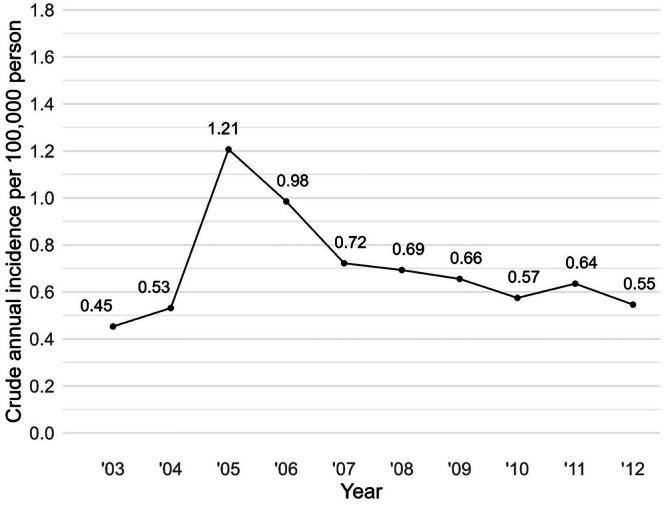
Annual incidence proportion of first-episode substance-induced psychosis in South Korea from 2003 to 2012.

The average annual incidence proportion of FE-SIP was 0.7 per 100,000 persons. Figure 1 illustrates the trend of FE-SIP incidence, showing that rates increased and peaked at 1.21 per 100,000 persons in 2005 before declining thereafter.

**Fig. 2:**
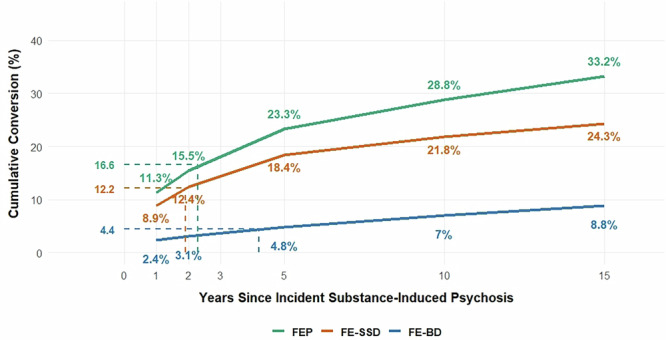
Cumulative conversion rate to Psychiatric disorders. Cumulative conversion rates from first-episode substance-induced psychosis to first-episode psychosis (FEP), schizophrenia spectrum disorders (FE-SSD), or bipolar disorders (FE-BD) are presented. Note: FE-BD First-Episode Bipolar Disorders, FEP First-Episode Psychosis, FE-SSD First-Episode Schizophrenia Spectrum Disorders.

Over a median follow-up of 9.3 years, (interquartile range [IQR]: 5.82–10.46 years), the cumulative conversion rates at 2, 5, 10, and 15 years were 15.5%, 23.3%, 28.8%, and 33.2%, respectively, for FEP; 12.4%, 18.4%, 21.8%, and 24.3% for FE-SSD; and 3.1%, 4.8%, 7.0%, and 8.8% for FE-BD (Fig. 2).

### Risk factors associated with conversion

In the multivariable models (Table 2), younger age (for ≤30, HR 1.65, 95% CI 1.20–2.27, *p* < 0.01; for ≤31–40, HR 1.38, 95% CI 1.10–1.73, *p* < 0.01), female sex (HR 1.38, 95% CI 1.13–1.69, *p* < 0.01), type of insurance (for self-employed, HR 1.30, 95% CI 1.06–1.59, *p* < 0.01; for medical aid, HR 1.45, 95% CI 1.06–2.00, *p* < 0.05), disability (HR 1.39, 95% CI 1.12–1.72, *p* < 0.01), other stimulants (HR 2.91, 95% CI 1.70–4.98, *p* < 0.0001), multidrug use (HR 1.79, 95% CI 1.29–2.49, *p* < 0.0005) and length of first hospitalization (HR 1.01, 95% CI 1.01–1.02, *p* < 0.0001) were all significantly associated with an increased conversion to FEP. For conversion to FE-SSD, type of insurance (HR 1.44, 95% CI 1.13–1.84, *p* < 0.0032), opioids (HR 2.29, 95% CI 1.08–4.87, *p* < 0.0314), other stimulant use (HR 3.10, 95% CI 1.69–5.69, *p* < 0.0005), multiple drug use (HR 1.78, 95% CI 1.20–2.64, *p* < 0.0040), and length of first hospitalization (HR 1.01, 95% CI 1.01–1.02, *p* < 0.0001) were significantly associated with an increased risk of conversion. In contrast, physical illnesses (HR 0.66, 95% CI 0.55–0.79, *p* < 0.0001) were significantly associated with a decreased risk of conversion. For conversion to FE-BD, younger age (for ≤30, HR 2.66, 95% CI 1.49–4.74, *p* < 0.001; for ≤31–40, HR 1.82, 95% CI 1.14–2.89, *p* < 0.0115), female sex (HR 1.75, 95% CI 1.22–2.52, *p* < 0.0024), and psychiatric service during the past year (HR 1.43, 95% CI 1.01–2.02, *p* < 0.05) were significantly associated with an increased conversion. Multiple drug use was not significantly associated with conversion to FE-BD (HR 1.76, 95% CI 0.98–3.18, *p* = 0.061). The results from the univariable Cox regression were generally similar, except for a significant finding on age in relation to the conversion to FE-SSD (Table S1).

**Table 2:** Multivariable Cox regression analysis of risk factors associated with conversion from first-episode substance-induced psychosis to first-episode psychosis, schizophrenia spectrum disorders or bipolar disorders.

Variables	Reference	FEP (*n* = 655)		FE-SSD (*n* = 493)		FE-BD (*n* = 162)	
		HR (95% CI)	*p*	HR (95% CI)	*p*	HR (95% CI)	*p*
Age							
≤30	51–60	1.65 (1.20; 2.27)	0.0022	1.35 (0.92; 1.98)	0.1235	2.66 (1.49; 4.74)	0.0010
31–40		1.38 (1.10; 1.73)	0.0055	1.26 (0.97; 1.63)	0.0556	1.82 (1.14; 2.89)	0.0115
41–50		1.19 (0.97; 1.45)	0.0911	1.10 (0.88; 1.38)	0.4100	1.54 (1.01; 2.34)	0.0467
Sex							
Female	Male	1.38 (1.13; 1.69)	0.0016	1.24 (0.98; 1.57)	0.0718	1.75 (1.22; 2.52)	0.0024
Type of insurance							
Self-employed insured	Employee insured	1.30 (1.06; 1.59)	0.0129	1.44 (1.13; 1.84)	0.0032		
Medical aid		1.45 (1.06; 2.00)	0.0215	1.75 (1.19; 2.55)	0.0040		
Health insurance quintiles							
Q2	Q1 (highest)	1.15 (0.73; 1.80)	0.5557	1.19 (0.67; 2.09)	0.5549		
Q3		1.09 (0.72; 1.66)	0.6779	1.35 (0.80; 2.27)	0.2659		
Q4		1.17 (0.76; 1.79)	0.4812	1.32 (0.77; 2.26)	0.3118		
Q5 (lowest)		1.44 (0.91; 2.28)	0.1219	1.53 (0.86; 2.73)	0.1478		
Unknown		0.70 (0.24; 2.05)	0.5171	0.89 (0.25; 3.11)	0.8548		
Psychiatric service during past 1-yr							
Yes	No	1.12 (0.93; 1.35)	0.2406			1.43 (1.01; 2.02)	0.0432
Physical illnesses							
Yes	No	0.72 (0.61; 0.85)	<0.0001	0.66 (0.55; 0.79)	<0.0001		
Disability							
Yes	No	1.39 (1.12; 1.72)	0.0028	1.45 (1.14; 1.84)	0.0026		
Types of substance							
Opioids	Alcohol	1.86 (0.92; 3.77)	0.0851	2.29 (1.08; 4.87)	0.0314	0.89 (0.12; 6.39)	0.9082
Cannabinoids		----	----	----	----	----	----
Sedatives		1.46 (0.60; 3.58)	0.4059	1.75 (0.64; 4.77)	0.2739	0.83 (0.11; 6.05)	0.8529
Cocaine		----	----	----	----	----	----
Other stimulants		2.91 (1.70; 4.98)	<0.0001	3.10 (1.69; 5.69)	<0.0005	2.49 (0.79; 7.84)	0.1197
Hallucinogens		1.15 (0.61; 2.17)	0.6642	1.40 (0.72; 2.73)	0.3199	0.41 (0.06; 2.96)	0.3783
Tobacco		1.38 (0.68; 2.80)	0.3697	1.43 (0.63; 3.23)	0.3883	1.11 (0.27; 4.54)	0.8798
Volatile solvents		1.33 (0.62; 2.84)	0.4690	1.90 (0.88; 4.10)	0.1031	----	----
Multiple drug use		1.79 (1.29; 2.49)	0.0005	1.78 (1.20; 2.64)	0.0040	1.76 (0.98; 3.18)	0.0606
Length of first hospitalization	1.01 (1.01; 1.02)	<0.0001	1.01 (1.01; 1.02)	<0.0001			

*CI* Confidence Interval, *FE-BD* First-Episode Bipolar Disorders, *FEP* First-Episode Psychosis, *FE-SIP* First-Episode Substance-Induced Psychosis, *FE-SSD* First-Episode Schizophrenia Spectrum Disorders, *HR* Hazard Ratio, Q Quintile.

### All-cause mortality

**Fig. 3:**
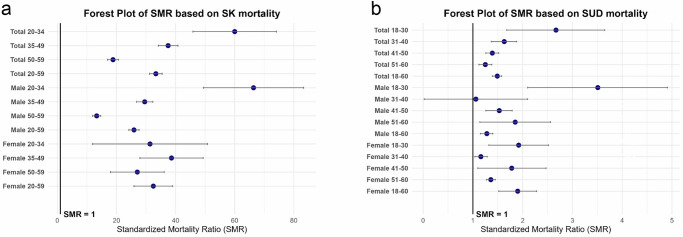
Standardized mortality ratios for individuals with first-episode substance-induced psychosis. Standardized mortality ratios (SMRs) were calculated from the index date to December 31, 2017. **a** Comparison against the total South Korean population aged 20–59 (mid-2015 to mid-2016). **b** Comparison against individuals aged 18–60 with a primary diagnosis of substance use disorder, identified between 2003 and 2012 and followed from the index date to December 31, 2017. Error bars indicate 95% confidence intervals (CIs). Note: FE-SIP First-Episode Substance-Induced Psychosis, SMR Standardized Mortality Ratio, SK South Korean, SUD Substance Use Disorders.

Compared to the general Korean population, SMR in individuals with FE-SIP was 33.33 (95% CI 31.20–35.46). This risk was particularly high among individuals aged 20–34 years (SMR 60.00, 95% CI 45.85–74.16), among male aged 20–34 (SMR 66.38, 95% CI 49.44–83.32), and among female aged 35–49 (SMR 38.62, 95% CI 27.92–49.33) (Fig. 3 and Table S2). Using individuals with SUD as the reference population, the SMR among individuals aged 18–60 years remained elevated with an overall SMR of 1.49 (95% CI 1.39–1.58). The SMR was higher among male aged 18–30 (SMR 3.51) and among female aged 41–50 (SMR 1.92) (Fig. 3 and Table S3).

## Discussion

Current evidence suggests that individuals who experience FE-SIP are at an increased risk of developing independent psychotic disorders. There is little research on its incidence, long-term outcome on the conversion rate and its risk factors, and mortality in Asia regions. Using nationwide registry data in SK, we estimated annual incidence of FE-SIP, and conversion rates to FE-SSD or FE-BD and mortality with up to 15-years of follow-up. Annual incidence proportion was relatively low, with modest to moderate conversion rates of 24.3% for FE-SSD and 8.8% for FE-BD at the end of follow-up. However, Individuals with FE-SIP exhibited a strikingly higher SMR than the general population of SK.

In our study population, alcohol was the most predominantly implicated substance in our FE-SIP cohort (92.91%), a rate substantially higher than those reported in Western studies (34.1–56.6%)^3,17^. This may reflect a relatively low proportion of other SIP cases. Or it may be associated with high prevalence of alcohol use disorder in Korea^22,23^. Several studies have suggested that the relatively high prevalence of AUD in SK is closely related to sociocultural drinking norms and a permissive drinking culture. Alcohol consumption in SK is strongly embedded in social interactions, including workplace gatherings, celebrations, and interpersonal bonding, which encourages frequent and heavy drinking^24^. Moreover, the per-capita alcohol consumption in SK (10.2 L per year) has been reported to exceed the global average^25^ The pathophysiology of alcohol-induced psychosis (AIP) is complex and may arise through multiple pathways, including direct toxic effects of alcohol^26,27^, effects of alcohol on neurotransmitters such as dopamine, gamma-aminobutyric acid and glutamate in relation to withdrawal^28,29^, and longer-term neuropsychiatric consequences of chronic use^30^. Unlike Western settings where cannabis-related psychosis predominates, AIP represents a clinically significant pathway to psychosis in Korea. This highlights the need for context-specific early detection strategies, careful diagnostic assessment to avoid misclassification as primary psychotic disorders, and the integration of alcohol-focused preventive and therapeutic interventions.

In addition, the pattern for other substances differed substantially, with a very low proportion of cannabinoids and a relatively high proportion of other stimulants. Our reported incidence proportion was considerably lower than those in Western and other Asian studies^8,9^, and no increasing trends were observed over the 10-year period. A possible explanation is that although rising drug use among youth in SK is becoming a social concern, it may not necessarily translate into an increased incidence of SIP. Notably, cannabis use is strictly prohibited in SK. From a methodological perspective, our stringent washout of any prior SUD diagnoses to ensure diagnostic purity likely further reduced the observed incidence by excluding chronic users who may have been included in other studies. When compared with the conversion rates to SSD or SZ in Western studies using similar nationwide registry data, our findings (24.3% or 18.4% over 15-year or 5-year, respectively) were higher^8,17,31^ or lower^16^ than those reported in other studies. Notably, Alderson et al. examined conversion to SZ only. The relatively high conversion rates observed in our study may be related to the relatively high proportion of other stimulants. This interpretation may be supported by our finding that the “other stimulants” category showed the highest HR for conversion to both FEP and FE-SSD in the multivariable analysis. This is in sharp contrast with the Western finding that cannabis-induced psychosis is associated with the highest risk of progression to SZ^3,17,18,32^. As the specific stimulant composition cannot be verified from the available data, it is difficult to determine which substances among other stimulants contributed most to the higher conversion rates observed in the present study. This question should be examined in future studies. Our conversion rates to FE-BD (8.8% or 4.8% over 15-year or 5-year, respectively) falls within the reported international range, spanning from 4.5% over 6-year^16^ to 24.3% over 15-year^8^. Specifically, our 15-year estimate (8.8%) is higher than the 8.4% reported in a 20-year Danish registry study^17^. An important clinical implication is that because half of the cases of conversion to FE-SSD or FE-BD occurred around two years, early intervention services should be provided for at least two years to identify individuals who will ultimately develop an independent psychosis.

The risk factors associated with conversion to both FEP and FE-BD were younger age and female sex. Younger age is one of the risk factors consistently reported^17,32^. The finding that female sex is a risk factor for conversion to FE-BD aligns with previous studies^16,17^. Risk factors associated with conversion to both FEP and FE-SSD were type of insurance, disability, and prolonged first hospitalization. Associations with type of insurance and disability suggest that poor socioeconomic status could be a risk factor for the conversion to FE-SSD. The finding that prolonged hospitalization was a risk factor for conversion to FE-SSD is in line with previous study^31^. In particular, the negative association with physical illnesses was an unexpected finding. It is possible that the ongoing medical care necessitated by physical illnesses may have played a beneficial role. Interestingly, psychiatric service during past 1-year was only associated with the conversion to FE-BD. This finding is consistent with the study of Starzer et al.^17^.

Most importantly, among the types of substance, other stimulants had the highest HR for the conversion to FEP and FE-SSD. Common stimulants include amphetamine, methamphetamine, methylphenidate, phentermine, and phendimetrazine. All these drugs are amphetamine analogs and reverse both vesicular monoamine transporter 2 and the dopamine transporter, thereby increasing synaptic concentrations of dopamine in the mesolimbic and the mesocortical pathways which in turn may precipitate psychosis^33^. In SK, prescriptions of medical narcotic appetite suppressants in Korea are predominantly issued to females (94%), with the highest prescription rates in the 30–40 age group^34^. In addition, numerous cases of psychosis induced by these drugs have been reported in SK^11–15^. An important question is the extent to which appetite suppressants contribute to the proportion of other stimulants-induced psychosis. As the current claims-based data do not specify specific agents within the “other stimulants” category, this hypothesis should be investigated in future studies. On the other hand, it is notable that AIP showed the lowest hazard ratio for conversion to FEP and FE-SSD compared to other substances. This finding suggests that AIP may represent a clinically more favorable and potentially modifiable pathway in the progression to more severe illnesses. However, these interpretations should be taken cautiously given that the sample was predominantly composed of AIP cases (89.97%) with very few cases of other substance-induced psychoses (11.03%). Overall, this finding suggests that when prescribing stimulants, clinicians should be mindful of the risk of FE-SIP and potential subsequent conversion to FE-SSD and should provide appropriate warnings and education to patients and caregivers.

Interestingly, we observed that HRs for FE-BD were 0.89, 0.83 and 0.41 for opioids, sedatives and hallucinogens respectively compared with alcohol as the reference group. This is consistent with previous findings showing that the conversion risk associated with other substances is lower than that associated with alcohol^17^. These relative risks underscore that alcohol-induced episodes may carry a higher risk of conversion to FE-BD than episodes induced by many other substances. This emphasizes the importance of monitoring for bipolarity in patients with alcohol-induced FE-SIP.

Lastly, our finding of an elevated SMR is consistent with two Danish nationwide register-based studies that SIP was strongly associated with an increased risk of suicide attempt^4^ and mortality^19^. In particular, it was interesting to observe a gender disparity in the age bands with the highest SMRs. Although suicidal ideation and suicide attempts among individuals with SUD are more common in females^35^, suicidal deaths related to SUD are more frequent in males^36^. In this respect, close monitoring and the provision of appropriate psychosocial interventions appear critical to reduce this excess mortality among individuals with FE-SIP, especially young men. Ultimately, the clinical significance of FE-SIP extends beyond diagnostic transition to encompass severe life-threatening outcomes.

This study has several limitations that should be considered when interpreting the findings. First, only a 1-year washout period may not have been sufficient to exclude all preexisting FE-SIP cases. However, extending this period was not feasible because of limited access to the data. Second, because we applied a stringent approach by excluding individuals with SUD during the washout period, the incidence rate of FE-SIP may have been substantially underestimated. The true incidence of SIP in SK could be much higher.as our estimate reflects only a very specific subpopulation of “SUD-naive” individuals. In addition, the limited number of conversion events in certain categories may have decreased the statistical power to detect subtle risk factors for diagnostic transition. Third, although this study was designed to investigate the broad category of SIP, the overwhelming predominance of alcohol-related cases (89.97%) limits the generalizability of the findings to other substances. Consequently, the results primarily reflect the clinical characteristics of an alcohol-predominant sample, and should be interpreted with caution when applied to other forms of SIP. Lastly, the registry data lack information on potentially important confounders, such as medication data, education level, employment status, and the precise quantity or frequency of substance misuse. Despite these caveats, the present study has notable strengths. It is the first investigation of FE-SIP in SK and includes comprehensive outcomes such as conversion rate and SMR. In addition, our findings on conversion provide an empirical basis for psychoeducation and close follow-up of individuals with FE-SIP.

In conclusion, in this nationwide cohort study, we found that the incidence of FE-SIP in SK was low, yet the risk of subsequent conversion to FE-SSD or FE-BD was substantial, particularly within the first two years of follow-up. Younger age, female sex, disability, prolonged first hospitalization, and exposure to stimulants were significant predictors of conversion. Importantly, individuals with FE-SIP exhibited markedly elevated mortality, especially among young males, underscoring the urgent need for close clinical monitoring and targeted interventions. Our findings highlight the importance of early detection, psychoeducation, and integrated care strategies tailored to individuals with FE-SIP to reduce the risk of conversion to psychosis and premature death.

## Supplementary information

Supplementary information

## Data Availability

Access to individual participant data (IPD) is strictly prohibited from public deposition due to legal and ethical obligations under the Korean National Health Insurance Service NHIS data governance. De-identified IPD access is strictly restricted for scientifically valid purposes. Researchers must submit a formal application for this access through the NHIS Big Data Analysis Center. The official data request page is available at: https://nhiss.nhis.or.kr/bd/ab/bdaba021eng.do The analytic code used to produce the results of this study will be made available upon request to the corresponding author to facilitate research transparency and reproducibility.

## References

[1] World Health Organization. *International Statistical Classification of Diseases and Related Health Problems, 10th revision*. (World Health Organization, 1992). 10.2471/BLT.25.294650 PMC13531092 42683092

[2] Fusar-Poli, P. et al. Diagnostic stability of ICD/DSM first episode psychosis diagnoses: meta-analysis. *Schizophr. Bull.* **42**, 1395–1406 (2016). 26980142 10.1093/schbul/sbw020 PMC5049518

[3] Murrie, B., Lappin, J., Large, M. & Sara, G. Transition of substance-induced, brief, and atypical psychoses to schizophrenia: a systematic review and meta-analysis. *Schizophr. Bull.* **46**, 505–516 (2020). 31618428 10.1093/schbul/sbz102 PMC7147575

[4] Munch, S. D., Madsen, T., Nordentoft, M., Erlangsen, A. & Hjorthøj, C. Association between substance-induced psychosis and suicide attempt: a Danish nation-wide register-based study. *Addiction* **118**, 2440–2448 (2023). 37574563 10.1111/add.16311

[5] Weibell, M. A. et al. Treated incidence and baseline characteristics of substance induced psychosis in a Norwegian catchment area. *BMC Psychiatry* **13**, 319 (2013). 24279887 10.1186/1471-244X-13-319 PMC4222718

[6] Rognli, E. B. et al. Annual incidence of substance-induced psychoses in Scandinavia from 2000 to 2016. *Psychol. Med.* **53**, 5246–5255 (2023). 35983644 10.1017/S003329172200229X PMC10476053

[7] Hjorthøj, C., Larsen, M. O., Starzer, M. S. & Nordentoft, M. Annual incidence of cannabis-induced psychosis, other substance-induced psychoses and dually diagnosed schizophrenia and cannabis use disorder in Denmark from 1994 to 2016. *Psychol. Med.* **51**, 617–622 (2021). 31839011 10.1017/S0033291719003532

[8] Chen, W. L., Hsieh, C. H., Chang, H. T., Hung, C. C. & Chan, C. H. The epidemiology and progression time from transient to permanent psychiatric disorders of substance-induced psychosis in Taiwan. *Addict. Behav.* **47**, 1–4 (2015). 25841087 10.1016/j.addbeh.2015.02.013

[9] Aggarwal, M., Banerjee, A., Singh, S. M., Mattoo, S. K. & Basu, D. Substance-induced psychotic disorders: 13-year data from a de-addiction centre and their clinical implications. *Asian J. Psychiatr.* **5**, 220–224 (2012). 22981049 10.1016/j.ajp.2011.11.008

[10] Kwon, N. J. & Han, E. A commentary on the effects of methamphetamine and the status of methamphetamine abuse among youths in South Korea, Japan, and China. *Forensic Sci. Int.* **286**, 81–85 (2018). 29567545 10.1016/j.forsciint.2018.02.022

[11] Jo, H. S., Wang, S. M. & Kim, J. J. Recurrent psychosis after phentermine administration in a young female: a case report. *Clin. Psychopharmacol. Neurosci.* **17**, 130–133 (2019). 30690949 10.9758/cpn.2019.17.1.130 PMC6361044

[12] Kim, M. D. et al. T64. Two cases of brief affective psychosis induced by diet aids. *Schizophr. Bull.* **45**, S228–S229 (2019).

[13] Kwak, S., Youn, T., Lee, N. Y., Chung, I. W. & Kim, S. H. Psychotic disorder induced by appetite suppressants, phentermine or phendimetrazine: a case series study. *Korean J. Biol. Psychiatry* **24**, 134–141 (2017).

[14] Kwon, S. J., Yang, J. C., Park, T. W. & Chung, Y. C. Appetite suppressor induced psychosis. *Clin. Psychopharmacol. Neurosci.* **8**, 170–174 (2010).

[15] Yun, J. A., Park, W. R., Yu, J. C. & Choi, K. S. A case of phendimetrazine induced-psychotic disorder and dependence. *J. Korean Neuropsychiatr. Assoc.* **52**, 402–405 (2013).

[16] Rognli, E. B., Heiberg, I. H., Jacobsen, B. K., Høye, A. & Bramness, J. G. Transition from substance-induced psychosis to schizophrenia spectrum disorder or bipolar disorder. *Am. J. Psychiatry* **180**, 437–444 (2023). 37132221 10.1176/appi.ajp.22010076

[17] Starzer, M. S. K., Nordentoft, M. & Hjorthøj, C. Rates and predictors of conversion to schizophrenia or bipolar disorder following substance-induced psychosis. *Am. J. Psychiatry* **175**, 343–350 (2018). 29179576 10.1176/appi.ajp.2017.17020223

[18] Kendler, K. S., Ohlsson, H., Sundquist, J. & Sundquist, K. Prediction of onset of substance-induced psychotic disorder and its progression to schizophrenia in a Swedish national sample. *Am. J. Psychiatry* **176**, 711–719 (2019). 31055966 10.1176/appi.ajp.2019.18101217 PMC6718312

[19] Hjorthøj, C., Madsen, T., Starzer, M., Erlangsen, A. & Nordentoft, M. Mortality in substance-induced psychosis: a register-based national cohort study. *Addiction* **116**, 3515–3524 (2021). 34105214 10.1111/add.15598

[20] Jang, H., Lee, W., Kim, Y.-O. & Kim, H. Suicide rate and social environment characteristics in South Korea: the roles of socioeconomic, demographic, urbanicity, general health behaviors, and other environmental factors on suicide rate. *BMC Public Health* **22**, 410 (2022). 35227243 10.1186/s12889-022-12843-4 PMC8887086

[21] Eun, S. J. Avoidable, amenable, and preventable mortalities in South Korea, 2000–2017: Age-period-cohort trends and impact on life expectancy at birth. *Soc. Sci. Med.* **237**, 112482 (2019). 31408768 10.1016/j.socscimed.2019.112482

[22] Ministry of Health and Welfare. *The 2021 Survey on Mental Health in Korea* (Ministry of Health and Welfare, 2021).

[23] World Health Organization. *Global status report on alcohol and health and treatment of substance use disorders* (World Health Organization, 2024).

[24] Ko, S. & Sohn, A. Behaviors and culture of drinking among Korean people. *Iran. J. Public Health* **47**, 47–56 (2018). 30186812 PMC6124142

[25] World Health Organization. *Global status report on alcohol and health 2018* (World Health Organization, 2018).

[26] Soyka, M., Dresel, S., Horak, M., Rüther, T. & Tatsch, K. PET and SPECT findings in alcohol hallucinosis: case report and super-brief review of the pathophysiology of this syndrome. *World J. Biol. Psychiatry* **1**, 215–218 (2000). 12607218 10.3109/15622970009150594

[27] Soyka, M., Koch, W. & Tatsch, K. Thalamic hypofunction in alcohol hallucinosis: FDG PET findings. *Psychiatry Res.* **139**, 259–262 (2005). 16043330 10.1016/j.pscychresns.2005.05.009

[28] Day, E. & Daly, C. Clinical management of the alcohol withdrawal syndrome. *Addiction* **117**, 804–814 (2022). 34288186 10.1111/add.15647

[29] McCunn, P., Chen, X., Gimi, B., Green, A. I. & Khokhar, J. Y. Glutamine and GABA alterations in cingulate cortex may underlie alcohol drinking in a rat model of co-occurring alcohol use disorder and schizophrenia: an 1H-MRS study. *Schizophrenia* **8**, 67 (2022). 35999232 10.1038/s41537-022-00272-6 PMC9399110

[30] Skryabin, V. Y., Martinotti, G., Franck, J. & Zastrozhin, M. S. Acute alcoholic hallucinosis: a review. *Psychopathology* **56**, 383–390 (2023). 36657433 10.1159/000528573

[31] Alderson, H. L. et al. Risk of transition to schizophrenia following first admission with substance-induced psychotic disorder: a population-based longitudinal cohort study. *Psychol. Med.* **47**, 2548–2555 (2017). 28464965 10.1017/S0033291717001118

[32] Niemi-Pynttäri, J. A. et al. Substance-induced psychoses converting into schizophrenia: a register-based study of 18,478 Finnish inpatient cases. *J. Clin. Psychiatry* **74**, e94–e99 (2013). 23419236 10.4088/JCP.12m07822

[33] Fiorentini, A. et al. Substance-induced psychoses: an updated literature review. *Front. Psychiatry* **12**, 694863 (2021). 35002789 10.3389/fpsyt.2021.694863 PMC8732862

[34] Oh, K. S. & Han, E. Analysis of prescription trends for narcotic appetite suppressants: utilizing the narcotics information management system. *Yonsei Med. J.* **65**, 480–487 (2024). 39048324 10.3349/ymj.2023.0335 PMC11284301

[35] Leza, L., Haro, B., López-Goñi, J. J. & Fernández-Montalvo, J. Substance use disorder and lifetime suicidal behaviour: a scoping review. *Psychiatry Res.* **334**, 115830 (2024). 38432115 10.1016/j.psychres.2024.115830

[36] Levola, J., Laine, R. & Pitkänen, T. In-patient psychiatric care and non-substance-related psychiatric diagnoses among individuals seeking treatment for alcohol and substance use disorders: associations with all-cause mortality and suicide. *Br. J. Psychiatry* **221**, 386–393 (2022). 35164892 10.1192/bjp.2022.20

