# Supplementary material for: Predictors of conversion to psychosis and mortality in first-episode substance-induced psychosis: a nationwide register-based study in South Korea
Source: Schizophrenia (Heidelb). 2026 May 6;12(1):59. doi: 10.1038/s41537-026-00760-z (PMC13338437; doi:10.1038/s41537-026-00760-z)
Supplement: Supplementary file 1 — Supplementary information [file 41537_2026_760_MOESM1_ESM.docx]

National Health Insurance Service Health Examinee Cohort DB

during 2003 and 2012

Individuals with the main Dx of F1x.5 (N = 12,409)

Total N = 7,817

• Inpatients: N = 5,367

• Outpatients with three consecutive main Dx of F1x.5: N = 2,450

- Outpatients with fewer than three consecutive main Dx of F1x.5 (N = 4,592)
- Individuals with a history of F10–F19, F00–F09, F20–F29, F30.x, F31.x, F32.3, or F33.3 Dx in the past 1-year: N = 4,863
- Age at cohort entry ≤ 18 or ≥ 60: N = 700
- Missing region value: N = 4
- Individuals with both F1x.5 and F20–F29 as main Dx at cohort entry in different hospitals and later found to have a greater or equal number of consecutive F20–F29 Dx than F1x.5 during the 5-year follow-up: N = 6

Final cohort

N = 2,244

Non-converter

N = 1,723

Converter to FE-BD

N = 107

Converter to FE-SSD

N = 414

**Figure S1.** **Flow chart depicting the process of obtaining the final study populations**

Note: Dx, Diagnosis; FE-BD, First-Episode Bipolar Disorders; FE-SSD, First-Episode Schizophrenia Spectrum Disorders.

**Table S1. Univariable Cox regression analysis of risk factors associated with conversion from first-episode substance-induced psychosis to** **first-episode psychosis, schizophrenia spectrum disorders or bipolar disorders**

| **Variables** | **Reference** | **FEP (n = 655)** | | **FE-SSD (n = 493)** | | **FE-BD (n = 162)** | |
| --- | --- | --- | --- | --- | --- | --- | --- |
|  |  | **HR (95% CI)** | ***p*** | **HR (95% CI)** | ***p*** | **HR (95% CI)** | ***p*** |
| Age |  |  |  |  |  |  |  |
| ≤ 30 | 51-60 | 2·01 (1·50; 2·70) | <·0001 | 1·66 (1·17; 2·36) | 0·0047 | 3·43 (1·98; 5·94) | <·0001 |
| 31-40 |  | 1·52 (1·22; 1·90) | 0·0002 | 1·42 (1·10; 1·82) | 0·0062 | 1·94 (1·22; 3·07) | 0·0048 |
| 41-50 |  | 1·30 (1·07; 1·58) | 0·0094 | 1·23 (0·98; 1·53) | 0·0698 | 1·57 (1·03; 2·39) | 0·0362 |
| Sex |  |  |  |  |  |  |  |
| Female | Male | 1·48 (1·23; 1·78) | <·0001 | 1·27 (1·02; 1·59) | 0·0364 | 2·20 (1·57; 3·09) | <·0001 |
| Type of insurance |  |  |  |  |  |  |  |
| Self-employed insured | Employee insured | 1·28 (1·05; 1·56) | 0·0139 | 2·28 (1·77; 2·93) | <·0001 | 1·19 (0·78; 1·81) | 0·4286 |
| Medical aid |  | 1·92 (1·55; 2·38) | <·0001 | 1·45 (1·14; 1·84) | 0·0023 | 0·95 (0·66; 1·37) | 0·7734 |
| Health insurance quintiles |  |  |  |  |  |  |  |
| Q2 | Q1 (highest) | 1·22 (0·78; 1·91) | 0·3854 | 1·29 (0·74; 2·28) | 0·3719 | 1·11 (0·53; 2·30) | 0·7871 |
| Q3 |  | 1·17 (0·78; 1·77) | 0·4521 | 1·48 (0·88; 2·48) | 0·1420 | 0·68 (0·34; 1·37) | 0·2810 |
| Q4 |  | 1·27 (0·83; 1·94) | 0·2695 | 1·50 (0·88; 2·56) | 0·1331 | 0·90 (0·44; 1·83) | 0·7657 |
| Q5 (lowest) |  | 1·94 (1·30; 2·90) | 0·0013 | 2·39 (1·44; 3·98) | 0·0008 | 1·21 (0·62; 2·37) | 0·5776 |
| Unknown |  | 0·86 (0·30; 2·47) | 0·7842 | 1·03 (0·30; 3·54) | 0·9601 | 0·59 (0·08; 4·59) | 0·6133 |
| Region |  |  |  |  |  |  |  |
| Urban | Rural | 0·85 (0·68; 1·06) | 0·1527 | 0·81 (0·63; 1·05) | 0·1102 | 0·98 (0·61; 1·58) | 0·9294 |
| Psychiatric service during past 1-yr | |  |  |  |  |  |  |
| Yes | No | 1·17 (0·98; 1·40) | 0·0851 | 1·03 (0·83; 1·27) | 0·7895 | 1·66 (1·19; 2·32) | 0·0028 |
| Physical illnesses | |  |  |  |  |  |  |
| Yes | No | 0·66 (0·57; 0·77) | <·0001 | 0·61 (0·51; 0·73) | <·0001 | 0·78 (0·58; 1·07) | 0·1199 |
| Disability |  |  |  |  |  |  |  |
| Yes | No | 1·56 (1·28; 1·90) | <·0001 | 1·66 (1·33; 2·08) | <·0001 | 1·23 (0·80; 1·91) | 0·3416 |
| Types of substance |  |  |  |  |  |  |  |
| Opioids | Alcohol | 2·13 (1·06; 4·28) | 0·0340 | 2·49 (1·18; 5·27) | 0·0165 | 1·05 (0·15; 7·49) | 0·9626 |
| Cannabinoids |  | ---- | ---- | ---- | ---- | ---- | ---- |
| Sedatives |  | 1·67 (0·69; 4·03) | 0·2531 | 1·76 (0·66; 4·71) | 0·2608 | 1·39 (0·20; 9·96) | 0·7417 |
| Cocaine |  | ---- | ---- | ---- | ---- | ---- | ---- |
| Other stimulants |  | 3·13 (1·84; 5·32) | <·0001 | 3·19 (1·76; 5·82) | 0·0001 | 2·92 (0·93; 9·17) | 0·0668 |
| Hallucinogens |  | 1·31 (0·70; 2·44) | 0·4019 | 1·54 (0·80; 2·98) | 0·2007 | 0·55 (0·08; 3·96) | 0·5566 |
| Tobacco |  | 1·12 (0·56; 2·26) | 0·7467 | 1·10 (0·49; 2·47) | 0·8115 | 1·18 (0·29; 4·79) | 0·8132 |
| Volatile solvents |  | 1·61 (0·76; 3·39) | 0·2104 | 2·13 (1·01; 4·49) | 0·0478 | ---- | ---- |
| Multiple drug use |  | 2·28 (1·68; 3·10) | <·0001 | 2·04 (1·41; 2·96) | 0·0002 | 3·04 (1·75; 5·27) | <·0001 |
| Length of first hospitalization | | 1·01 (1·01; 1·02) | <·0001 | 1·01 (1·01; 1·02) | <·0001 | 1·01 (1·00; 1·02) | 0·284 |

Note: CI, Confidence Interval; FE-BD, First-Episode Bipolar Disorders; FEP, First-Episode Psychosis; FE-SIP, First-Episode Substance-Induced Psychosis; FE-SSD, First-Episode Schizophrenia Spectrum Disorders; HR, Hazard Ratio; Q, Quintile.

**Table S2. Standardized mortality ratios for individuals with first-episode substance-induced psychosis from index date to December 31, 2017, calculated using the reference mortality rates of total South Korean population aged 20–59** **between mid-2015 to mid-2016**

| **Total FE-SIP/reference:**  **Total SK population** | **Age band** | **FE-SIP population** | **Observed death** | **Expected death** | **SMR (95% CI)** |
| --- | --- | --- | --- | --- | --- |
| Total | 20-34 | 280 | 69 | 1·15 | 60·00 (45·85; 74·16) |
|  | 35-49 | 1157 | 488 | 13·02 | 37·48 (34·16; 40·81) |
|  | 50-59 | 745 | 383 | 20·33 | 18·84 (16·96; 20·73) |
|  | Total | 2182 | 940 | 28·21 | 33·33 (31·20; 35·46) |
| Male | 20-34 | 173 | 59 | 0·89 | 66·38 (49·44; 83·32) |
|  | 35-49 | 983 | 438 | 14·82 | 29·56 (26·79; 32·32) |
|  | 50-59 | 663 | 349 | 26·22 | 13·31 (11·91; 14·71) |
|  | Total | 1819 | 846 | 32·63 | 25·93 (24·18; 27·67) |
| Female | 20-34 | 107 | 10 | 0·32 | 31·35 (11·92; 50·78) |
|  | 35-49 | 174 | 50 | 1·29 | 38·62 (27·92; 49·33) |
|  | 50-59 | 82 | 34 | 1·26 | 27·05 (17·96; 36·15) |
|  | Total | 363 | 94 | 2·89 | 32·47 (25·91; 39·04) |

Note: CI, Confidence interval; FE-SIP, First-Episode Substance-Induced Psychosis; SK, South Korean; SMR, Standardized Mortality Ratio.

**Table S3. Standardized mortality ratios for individuals with first-episode substance-induced psychosis from index date to December 31, 2017,** **calculated using the reference mortality rates of individuals aged 18–60 with a primary diagnosis of substance use disorders identified between 2003 and 2012 and followed from their index date to December 31, 2017**

| **Total FE-SIP/ reference:**  **SUD** | **Age band** | **FE-SIP population** | **Observed death** | **Expected death** | **SMR (95% CI)** |
| --- | --- | --- | --- | --- | --- |
| Total | 18-30 | 153 | 28 | 10·51 | 2·67 (1·68; 3·65) |
|  | 31-40 | 469 | 157 | 96·47 | 1·63 (1·37; 1·88) |
|  | 41-50 | 919 | 424 | 305·24 | 1·39 (1·26; 1·52) |
|  | 51-60 | 703 | 358 | 286·81 | 1·25 (1·12; 1·38) |
|  | Total | 2244 | 967 | 649·88 | 1·49 (1·39; 1·58) |
| Male | 18-30 | 88 | 24 | 6·85 | 3·51 (2·10; 4·91) |
|  | 31-40 | 359 | 131 | 85·84 | 1·53 (1·26; 1·79) |
|  | 41-50 | 797 | 385 | 301·61 | 1·28 (1·15; 1·40) |
|  | 51-60 | 627 | 332 | 285·32 | 1·16 (1·04; 1·29) |
|  | Total | 1871 | 872 | 643·40 | 1·36 (1·27; 1·45) |
| Female | 18-30 | 65 | 4 | 3·77 | 1·06 (0·02; 2·10) |
|  | 31-40 | 110 | 26 | 14·04 | 1·85 (1·14; 2·56) |
|  | 41-50 | 122 | 39 | 20·32 | 1·92 (1·32; 2·52) |
|  | 51-60 | 76 | 26 | 14·60 | 1·78 (1·10; 2·47) |
|  | Total | 373 | 95 | 50·06 | 1·90 (1·52; 2·28) |

Note: CI, Confidence interval; FE-SIP, First-Episode Substance-Induced Psychosis; SMR, Standardized Mortality Ratio; SUD, Substance Use Disorders Without Psychosis.

**STROBE-checklist-for-cohort**

|  | Item No. | Recommendation | Page No. | Relevant text from manuscript |
| --- | --- | --- | --- | --- |
| Title and abstract | 1 | (a) Indicate the study’s design with a commonly used term in the title or the abstract | 1 | Title page |
|  |  | (b) Provide in the abstract an informative and balanced summary of what was done and what was found | 2-3 | Abstract |
| Research in context and Introduction | | | | |
| Background/rationale | 2 | Explain the scientific background and rationale for the investigation being reported | 3-5 | Research in context and introduction |
| Objectives | 3 | State specific objectives, including any prespecified hypotheses | 6 | Introduction |
| Methods | | | | |
| Study design | 4 | Present key elements of study design early in the paper | 6-7 | Method |
| Setting | 5 | Describe the setting, locations, and relevant dates, including periods of recruitment, exposure, follow-up, and data collection | 7 | Method |
| Participants | 6 | (a) Give the eligibility criteria, and the sources and methods of selection of participants. Describe methods of follow-up | 7 | Method |
|  |  | (b) For matched studies, give matching criteria and number of exposed and unexposed | NA | NA |
| Variables | 7 | Clearly define all outcomes, exposures, predictors, potential confounders, and effect modifiers. Give diagnostic criteria, if applicable | 7 | Method |
| Data sources/ measurement | 8* | For each variable of interest, give sources of data and details of methods of assessment (measurement). Describe comparability of assessment methods if there is more than one group | 6-7 | Method |
| Bias | 9 | Describe any efforts to address potential sources of bias | 8 | Method |
| Study size | 10 | Explain how the study size was arrived at | NA | NA |
| Quantitative variables | 11 | Explain how quantitative variables were handled in the analyses. If applicable, describe which groupings were chosen and why | 7-8 | Method |
| Statistical methods | 12 | (a) Describe all statistical methods, including those used to control for confounding | 8-9 | Method |
|  |  | (b) Describe any methods used to examine subgroups and interactions | 8-9 | Method |
|  |  | (c) Explain how missing data were addressed | NA | NA |
|  |  | (d) If applicable, explain how loss to follow-up was addressed | 8-9 | Method |
|  |  | (e) Describe any sensitivity analyses | 8-9 | Method |
| Results | | | | |
| Participants | 13* | (a) Report numbers of individuals at each stage of study—eg numbers potentially eligible, examined for eligibility, confirmed eligible, included in the study, completing follow-up, and analysed | 9-11 | Result |
|  |  | (b) Give reasons for non-participation at each stage | Figure S1 | Figure S1 |
|  |  | (c) Consider use of a flow diagram | Figure S1 | Figure S1 |
| Descriptive data | 14* | (a) Give characteristics of study participants (eg demographic, clinical, social) and information on exposures and potential confounders | 9-11 | Result |
|  |  | (b) Indicate number of participants with missing data for each variable of interest | NA | NA |
|  |  | (c) Summarise follow-up time (eg, average and total amount) | 9-11 | Result |
| Outcome data | 15* | Report numbers of outcome events or summary measures over time | 11 | Result |
| Main results | 16 | (a) Give unadjusted estimates and, if applicable, confounder-adjusted estimates and their precision (eg, 95% confidence interval). Make clear which confounders were adjusted for and why they were included | 10 | Result |
|  |  | (b) Report category boundaries when continuous variables were categorized | 10 | Result |
|  |  | (c) If relevant, consider translating estimates of relative risk into absolute risk for a meaningful time period | 10-11 | Result |
| Other analyses | 17 | Report other analyses done—eg analyses of subgroups and interactions, and sensitivity analyses | NA | NA |
| Discussion | | | | |
| Key results | 18 | Summarise key results with reference to study objectives | 11 | Discussion |
| Limitations | 19 | Discuss limitations of the study, taking into account sources of potential bias or imprecision. Discuss both direction and magnitude of any potential bias | 14 | Discussion |
| Interpretation | 20 | Give a cautious overall interpretation of results considering objectives, limitations, multiplicity of analyses, results from similar studies, and other relevant evidence | 14 | Discussion |
| Generalisability | 21 | Discuss the generalisability (external validity) of the study results | 14 | Discussion |
| Other information | | | | |
| Funding | 22 | Give the source of funding and the role of the funders for the present study and, if applicable, for the original study on which the present article is based | 3 | Abstract |
